# Real-time detection of acetone gas molecules at ppt levels in an air atmosphere using a partially suspended graphene surface acoustic wave skin gas sensor†

**DOI:** 10.1039/d3na00914a

**Published:** 2023-11-17

**Authors:** Haolong Zhou, Sankar Ganesh Ramaraj, Kaijie Ma, Md Shamim Sarker, Zhiqiang Liao, Siyi Tang, Hiroyasu Yamahara, Hitoshi Tabata

**Affiliations:** a Department of Electrical Engineering and Information Systems, Graduate School of Engineering, The University of Tokyo 7-3-1 Hongo, Bunkyo-ku Tokyo 113-8656 Japan tabata@g.ecc.u-tokyo.ac.jp yamahara@bioxide.t.u-tokyo.ac.jp ramaraj@g.ecc.u-tokyo.ac.jp; b Department of Bioengineering, Graduate School of Engineering, The University of Tokyo 7-3-1 Hongo Tokyo 113-8656 Japan

## Abstract

To improve the quality of modern life in the current society, low-power, highly sensitive, and reliable healthcare technology is necessary to monitor human health in real-time. In this study, we fabricated partially suspended monolayer graphene surface acoustic wave gas sensors (G-SAWs) with a love-mode wave to effectively detect ppt-level acetone gas molecules at room temperature. The sputtered SiO_2_ thin film on the surface of a black 36°YX-LiTaO_3_ (B-LT) substrate acted as a guiding layer, effectively reducing the noise and insertion loss. The G-SAWs exhibited enhanced gas response towards acetone gas molecules (800 ppt) in a real-time atmosphere. The high sensitivity of the G-SAW sensor can be attributed to the elasticity and surface roughness of the SiO_2_ film. In addition, the G-SAW sensor exhibited rapid response and recovery at room temperature. This study provides a potential strategy for diagnosing different stages of diabetes in the human body.

## Abstract 2

To improve the quality of modern life in the current society, low-power, highly sensitive, and reliable healthcare technology is necessary to monitor human health in real-time.

## Graphical abstract

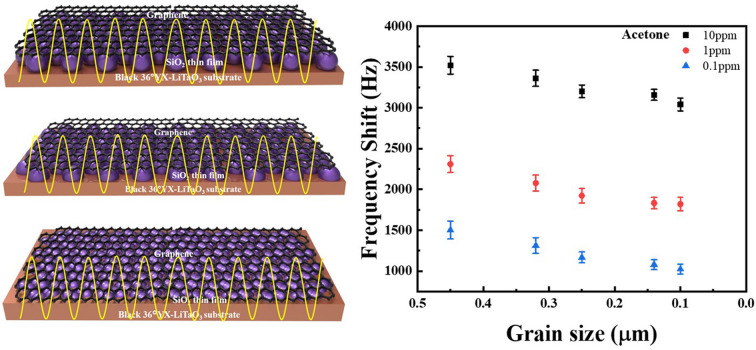

## 1 Introduction

With the development of modern urbanization, humans are struggling with numerous health and environmental problems. Owing to these issues, the lifespan and health condition of human beings have been gradually declining in recent years.^1–4^ To improve the lifespan and health conditions of human beings, an advanced device that can monitor their health conditions regularly in a real-time environment is required. Exhaled breath (EB) and skin gas analyses are effective approaches that can alter traditional medical diagnostic methods and revolutionize medical devices by providing timely reports and portable functions.^5–7^ Moreover, compared to traditional healthcare services, skin gas analysis is cheap, noninvasive, and can diagnose diseases in their early stages. Thus, the skin gas technique is promising and effective for the detection and diagnosis of various diseases by detecting biomarkers on the human skin. The skin gas analysis technique is considerably easier and more effective than the EB method for collecting skin gas molecules without any burden on patients. Diabetes is the primary cause of cardiovascular diseases and diabetic retinopathy. Recently, the number of people affected by diabetes has surged by approximately 500 million, making it a major global disease. The concentration of acetone emitted from the skin of diabetic patients exceeds approximately 77–97 parts per billion (ppb), which is quite low compared to exhaled breath (0.3–0.9 ppm).^8–10^ Therefore, real-time monitoring of the acetone concentration in human skin can be used as an effective diagnostic criterion for the detection of diabetes in its early stages. Hence, an efficient and meticulous design of an acetone gas sensor is required to detect parts per billion to parts per trillion (ppb–ppt) of gas molecules in the skin and exhaled breath. To identify biomarkers in human breath and skin, different types of gas sensors have been utilized, such as electrical resistance change semiconductors or metal oxides and electrochemical, optical, capacitance, calorimetric, and surface acoustic wave gas sensors.^11–20^ However, they are insufficient for detecting low concentrations of skin gas molecules.

Surface acoustic waves (SAWs) have received considerable attention in the field of gas sensing owing to their superior thermal stability, lower phase noise compared to crystal resonators, high quality, and superior temperature characteristics.^21–24^ The SAW resonator works on the surface, making it highly sensitive to external changes in the environment, such as temperature, gases, and humidity. Therefore, any disturbance or change on the surface of the sensing layer (*e.g.*, mass loading, electrical loading, and elastic loading) has a significant effect on the propagation of acoustic waves, resulting in changes in frequency, phase, and amplitude.^25^ However, the SAW sensor exhibited lower sensitivity and selectivity towards low-concentration gas molecules. Therefore, researchers have explored different technologies, such as lamp mode, Rayleigh mode, and Love mode SAW sensors, to improve the detection limit.^26–29^ Among the above-mentioned techniques, the Love-wave-mode SAW sensor is one of the preferred SAW gas sensors because of the effective guidance of the waves close to the sensor surface. When the Love wave propagates along the surface of the guide layer, the lower velocity guide layer produces an impedance mismatch at the boundary, which helps limit the Love wave energy to the surface rather than penetrating the main body of the material, resulting in a lower insertion loss than body wave propagation. Certain conditions are required to generate the Love-wave mode in the SAW sensor. In the Love wave mode, the shear velocity in the guiding layer should be smaller than the shear velocity in the substrate. It is important to choose an overlay material which has low shear velocity, low density, and low acoustic absorption. Fused silica (SiO_2_) satisfies these conditions for the existence of Love waves with excellent elastic and thermal properties compared to other materials (polymers, ZnO, *etc.*) besides low acoustic losses, excellent abrasion resistance to water, and chemical degradation.^30^ Hence, the Love-wave sensor with a SiO_2_ thin film as the guiding layer will achieve high sensitivity and low noise in chemical gas sensing.^31–35^

In this study, monolayer chemical vapor deposition (CVD) graphene Love-wave SAW sensors were fabricated for effective real-time detection of ppt-level acetone gas molecules. The SiO_2_ guiding layer deposited on the surface of the black 36°YX-LiTaO_3_ (B-LT) substrate effectively decreased the insertion losses and noise levels in the device. It is worth mentioning that a CVD graphene sensor with a 3 μm SiO_2_ guiding layer exhibited an excellent gas response as low as 800 ppt, which can detect skin gas molecules and meet the needs of diabetes monitoring in medical diagnosis applications. The enhanced gas response mechanism is briefly explained based on the roughness of the SiO_2_ film and the adsorption orientation.

## 2 Experimental

### 2.1 Device fabrication

An 80 nm (Cr/Au) thick interdigital transducer (IDT) pattern was fabricated using photolithography, followed by lift-off. We designed different distances (30, 60, and 90) between the IDT electrode and 30 pairs of reflective grids behind the IDT to improve the stability of the SAW device, as listed in Table 1. The design with a 32 μm period distance and 60 pairs of reflective grids demonstrated better stability than the other designs. The central sensing area was designed to be 4 × 4 mm which functioned as the gas-adsorption area.

**Table 1:** Structural parameters of the G-SAW device

SAW wavelength *λ*	32 μm
Acoustic aperture	4 mm
IDT's finger pair number	30, 60, 90
Reflector's electrode number	30
IDT thickness (Cr/Au)	80 nm

### 2.2 Deposition of the SiO_2_ layer

SiO_2_ layers with different thicknesses (1, 2, 3, and 4 μm) were deposited using magnetron sputtering technology. Before deposition of the SiO_2_ layer, the B-LT substrate was cleaned using a standard chemical process. The substrate was precisely placed above the target with a 50 mm distance between them. An RF power of approximately 80 W was used to deposit the SiO_2_ layer in an Ar atmosphere (20 sccm).

### 2.3 Graphene transfer process

**Fig. 1:**
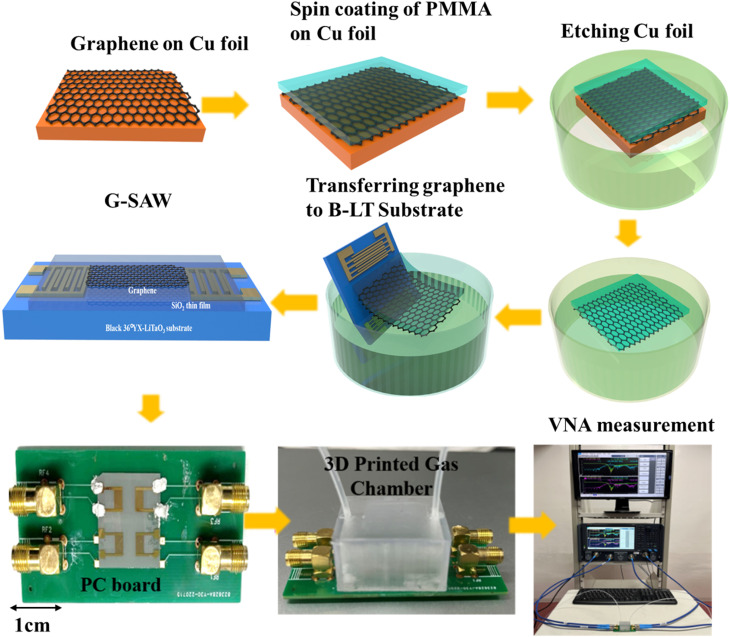
Schematic representation of fabrication and gas sensing measurement setup of the SAW graphene sensor with the 3D printed gas chamber.

Commercially available CVD graphene is utilized as a sensing layer to fabricate G-SAW sensor devices. Polymethylmethacrylate (PMMA) was spin-coated on a copper/graphene substrate and heated at 150 °C for 2 min. Furthermore, the copper foil was etched with oxygen plasma and 0.1 M of ammonium persulfate solution. The graphene was transferred to the SiO_2_ thin film/B-LT substrate as depicted in Fig. 1. The substrate was immersed in the acetone at 60 °C to remove the PMMA on the surface of the graphene.

### 2.4 Gas sensing measurement

We designed a printed circuit board (PCB) and 3D-printed gas chamber for gas-sensing measurements (Fig. 1). The response of the SAW sensor is defined as Δ*f* = *f*_s_ − *f*_0_, where *f*_s_ is the oscillation frequency of the sensor when exposed to the test gas and *f*_0_ is the center frequency of the sensor in the ambient environment, respectively. The oscillation frequency of the SAW sensor was recorded using a network analyzer (PNA-N5222B). The standard gas was diluted with N_2_ and the measurements were performed in an air atmosphere. The SAW waveform characteristics of the graphene were measured before and after the introduction of the target gas molecules. N_2_ gas molecules were purged into the chamber to recover the device to its original stage.

## 3 Results and discussion

**Fig. 2:**
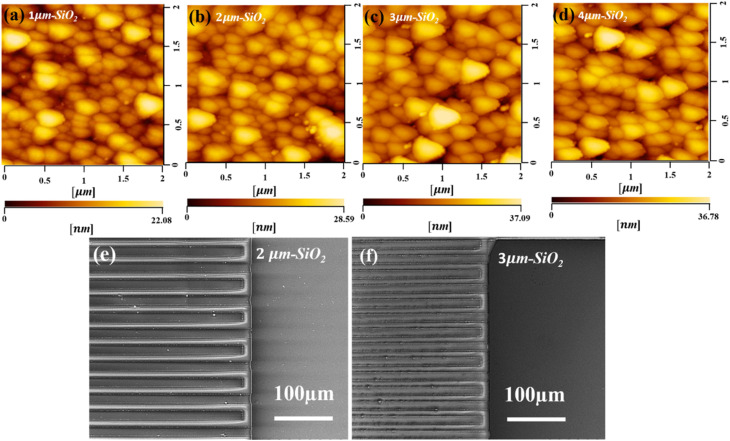
(a–d) AFM and (e and f) SEM analysis of the SiO_2_ layer on a black-36°YX LiTaO_3_ substrate with different thicknesses respectively.

**Fig. 3:**
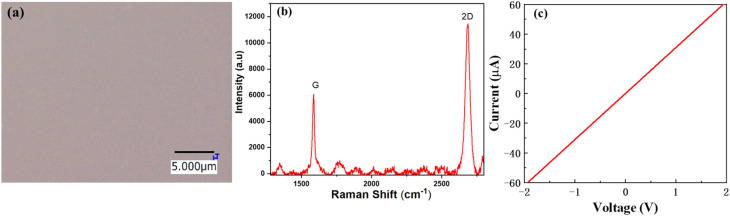
(a) Optical image of transferred CVD graphene on the SiO_2_/black-36°YX LiTaO_3_ substrate. (b) Raman spectrum of the CVD G-SAW sensor. (c) *I*–*V* characteristics of the fabricated G-SAW sensor.

The surface morphology was investigated using atomic force microscopy (AFM) to determine the surface roughness of the sputtered SiO_2_ thin films. Fig. 2(a–d) illustrate the 2D AFM images of deposited (1, 2, 3, and 4 μm) SiO_2_ guiding layers on the surface of the substrate, respectively. The AFM image indicates that the roughness of the film increased as the thickness of SiO_2_ increased. In addition, a considerable number of SiO_2_ nanoparticles were uniformly distributed on the surface, and the diameter of the nanoparticles increased with the SiO_2_ thickness. The uniformity of the prepared SiO_2_ films is illustrated in Fig. S1a,† the root mean square (RMS) roughness increased as the thickness of SiO_2_ increased from 3.9 to 5.5 nm. Fig. S1b† shows that the grain size decreased as the temperature increased. These results indicate that a uniform film was grown at higher temperatures. Fig. S2† shows an AFM image of a 3 μm SiO_2_ film deposited at different temperatures. The results indicated that temperature played a vital role in the formation of a uniform film and reduced the roughness of the film. The surface morphology of the sputtered SiO_2_ thin film was further analyzed using scanning electron microscopy (SEM), as shown in Fig. 2(e and f). The results indicated that the SiO_2_ thin film was uniformly deposited on the surface of the substrate and between the gaps of the IDT. The IDT pattern is completely covered with a SiO_2_ layer (3 μm) compared to 2 μm which can enhance and guide the Love mode surface acoustic wave to travel within the guiding layer. Fig. 3(a) shows an optical image of the graphene transferred onto the SiO_2_/B-LT substrate. The image shows that the graphene was successfully transferred without any cracks or voids. Raman analysis is an efficient technique for analyzing the optical properties, lattice structure, phonon modes, and electronic properties of a material. The Raman spectrum of the G-SAW device shows major peaks which correspond to the G band (1587 cm^−1^) and 2D band (2687 cm^−1^). The G band is attributed to the sp^2^-bonded carbon atoms in the graphite ring and the 2D band corresponds to the double-resonant Raman scattering of the two phonon emissions, respectively, and the intensity ratio 2*D*/*G* ≈ 2, indicating that the CVD graphene is a monolayer (Fig. 3(b)). In addition, the additional peaks, aside from the main peak, were substrate peaks attributed to B-LT. Initially, drain current (*I*_d_) and drain voltage (*V*_d_) measurements were performed on the fabricated G-SAW device using a semiconductor analyzer. The device exhibited ohmic conductance, as shown in Fig. 3(c).

**Fig. 4:**
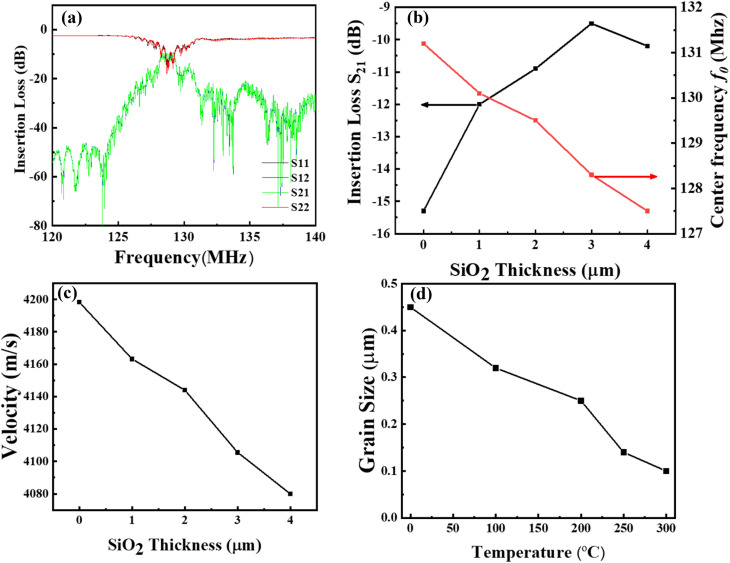
(a) Surface acoustic wave spectra of 3 μm deposited SiO_2_, (b) loss reduction and center frequency shift of different thicknesses of the deposited SiO_2_ layer, (c) Love wave propagation velocity *vs.* thickness of the SiO_2_ layer, (d). The temperature dependence of grain size corresponds to 3 μm SiO_2_.

The effect of SiO_2_ thickness of a guiding layer was analyzed using a vector network analyzer (VNA), as shown in Fig. 4(a–d). The results indicate that the noise of the SAW waveform is significantly reduced, with the center frequency (130 MHz to 129 MHz) and insertion loss (−15.7 dB to −12.4 dB) as depicted in Fig. 4(a) and S3.† This indicates that the acoustic energy can be easily captured on the surface by the guiding layer. In addition, the SiO_2_ guide layer with 3 μm shows further reduction in the noise level due to the acoustic energy aggregation, resulting in an increased insertion loss of −9.6 dB compared to other thicknesses. The reduction in insertion loss and noise may be due to the effective guidance of acoustic energy and the enhanced electromechanical coupling of elastic waves by the IDTs in the superposition.^36^ Strong electromechanical coupling can be achieved when the maximum acoustic energy is between the substrate and layer interface. In addition, the surface acoustic wave tends to propagate more in the waveguide layer; therefore, the acoustic energy is more concentrated in the waveguide layer and is less likely to leak into the substrate, which enhances the performance of the fabricated SAW sensor with high insertion loss.^30^ As shown in Fig. 4(b), a further increase in the thickness resulted in a decrease in the insertion loss, possibly due to the lower coupling effect. Fig. 4(c) shows the Love-wave propagation velocity as a function of the deposited thickness of the SiO_2_ guiding layer. The velocity decreases with an increase in the thickness of the SiO_2_ guiding layer since the shear wave velocity of the B-LT substrate has a higher shear velocity (4202 ms^−1^) than the SiO_2_ guiding layer (2850 ms^−1^) which reduces the acoustic attenuation and increases mass sensitivity in the Love wave mode SAW sensor.^32,37^Fig. 4(d) shows the grain size of the deposited SiO_2_ guiding layer deposited at different temperatures. The grain size of the film quickly decreased with increasing temperature, indicating the formation of a uniform film.

### 3.1 Investigation of the gas sensor

**Fig. 5:**
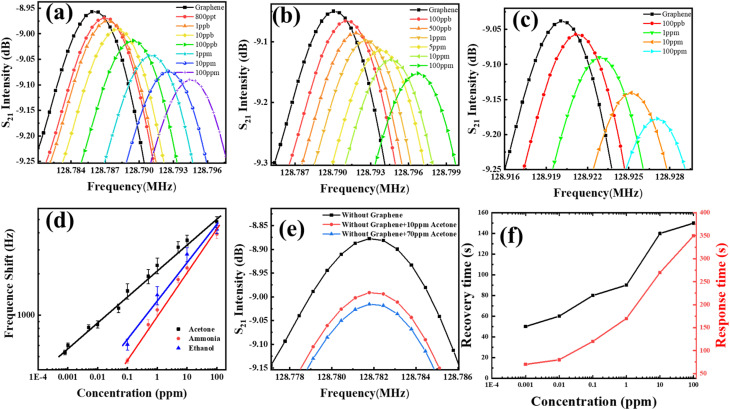
Gas sensing analysis of the G-SAW sensor: (a) acetone, (b) ammonia, (c) ethanol. (d) Comparative study of frequency shifts toward different concentrations of acetone, ammonia, and ethanol gas molecules. (e) Acetone gas sensing analysis without CVD graphene. (f) Recovery time of the G-SAW sensor in response to acetone, ethanol, and ammonia gas molecules.

Fig. 5(a–c) illustrate the center frequency response plots of the G-SAW sensor towards acetone, ethanol, and ammonia gas molecules, respectively, at room temperature and relative humidity (10–95%). The peak corresponds to the center frequencies *f*_0_ and *f*_s_ of the waveform under an ambient atmosphere and gas molecules. As evident from the sensing results, the positive frequency shift of Δ*f* gradually increases as the concentrations of gas molecules increase. Fig. S4† shows the dynamic frequency response curves of the G-SAW sensor towards acetone, ethanol, and ammonia gas molecules at different concentrations, which exhibited a linear relationship when the concentrations varied between 800 and 100 ppm, respectively. However, the G-SAW sensor showed a higher sensitivity towards acetone gas molecules than to other gas molecules (Fig. 5(d)). Fig. 5(e) shows the SAW devices without a CVD graphene sensing layer toward acetone molecules. The results revealed no shift in frequency even towards higher concentrations of acetone gas molecules. This demonstrates the role of graphene in sensing gas molecules in our experiments. Fig. S4† shows the effect of the temperature of the gas sensor toward acetone and ammonia gas molecules. The results reveal that the gas response of acetone gas increases monotonically up to 60 °C and decreases slightly above that temperature. However, ammonia exhibited a lower response than acetone gas molecules. Fig. S5† shows the response to acetone gas (100 ppm) under different humidity and temperature conditions. As humidity increases, water molecules are physisorbed on the surface of graphene, leading to a slight decrease in sensitivity. When the relative humidity was further increased, the multilayer water molecules were adsorbed. This results in a mass effect in the graphene SAW sensor. Fig. 5(f) shows the response and recovery curves of the acetone gas molecules at room temperature. The results indicated that acetone gas exhibited a quick response and recovery at very low concentrations. The acetone gas molecules take a long time to recover, which may be due to the stronger van der Waals (vdW) interactions between the graphene and acetone gas molecules. The observed sensitivity was significant compared to that of other recent SAW gas sensors (see Table 2).

**Table 2:** Comparison of acetone gas sensing: prior SAW sensors reported *vs.* current work^a^

Type	Sensing film	Temperature (°C)	Target gas	Concentration	Ref.
SAW	Ppy	RT	Acetone	5.5 ppm	38
SAW	Cu/SWCNTs	RT	Acetone	100 ppm	39
CR	NiO/ZnO	RT	Acetone	0.8 ppm	40
CR	ZnO/S, N: GQDs/PANI	RT	Acetone	5.5 ppm	41
CR	W_18_O_49_/Ti_3_C_2_Tx Mxene	RT	Acetone	0.17 ppm	42
SAW	QDs/Ppy	RT	Acetone	0.5 ppm	43
QCM	PDA-GO	RT	Acetone	400 ppm	44
SAW	Suspended graphene	RT	Acetone	0.008 ppm	This work

a CR – Chemo-resistive, RT – room temperature.

## 4 Discussion of the sensing mechanism

In this study, the G-SAW device showed a slight shift in the center frequency when exposed to gas molecules. This shift in the center frequency can be attributed to various phenomena, such as the elasticity effect, mass effect, and acoustoelectric effect, as shown in eqn (1) ^45,46^1
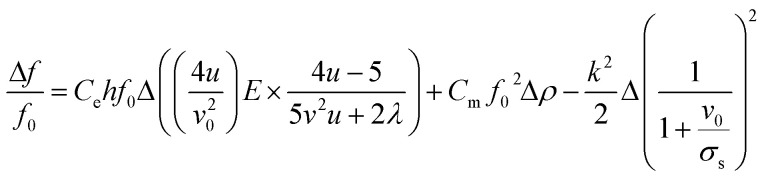
*f*_0_: fundamental frequency of the SAW sensor, *ρ*: the mass surface density of the sensitive coating (mass per unit area), *k*^2^ (9%): the electromechanical coupling coefficient, *v*_0_ (4200 ms^−1^): the undisturbed SAW velocity, *σ*_s_: the sheet conductivity of the sensitive layer, *E*: Young's modulus of the materials, *v*: Poisson's ratio of the materials, *u*: the shear modulus of elasticity, *C*_e_: the coefficients of elasticity, *C*_m_: the coefficients of elasticity, *C*_s_ (4 pF cm^−1^): the capacitance per unit length of the piezoelectric substrate.

**Fig. 6:**
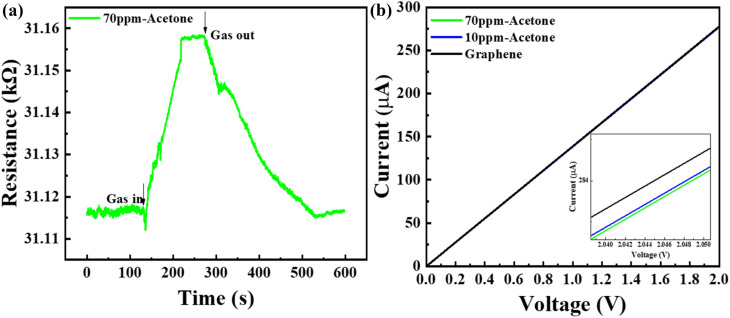
(a) Electrical resistance of the graphene film when exposed to 70 ppm acetone gas, (b) *I*–*V* curve of the graphene film when exposed to 70 ppm and 10 ppm acetone gas.

In our study, the shift in the center frequency (Δ*f*) may be related to the mass loading, elastic properties, or acoustic–electric interaction effects of the adsorbed gas molecules on the graphene surface. The acoustic effect is related to the changes in the electrical conductivity and capacitance of the sensing layer. Hence, the acoustic–electric interaction of the fabricated graphene sensor was exposed to acetone gas molecules at room temperature. The change in the resistance was monitored separately using gold electrodes with and without gas molecules. The initial resistance of the graphene sensor was found to be approximately 31.11 kΩ and the resistance slightly increased when exposed to acetone gas molecules (Fig. 6(a)). The calculated value indicated that the factor 
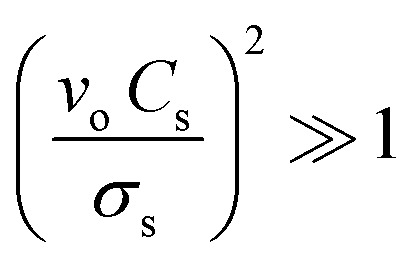
, which shows that the contribution of the acoustic interaction is negligible for the graphene/SiO_2_/B-LT sensor device. In addition, the current *vs.* voltage measurements were augmented by the aforementioned results, as depicted in Fig. 6(a). Hence, the contribution of the gas response may be attributed to other effects. The mass-loading effect causes a shift in the center frequency towards the negative side, and the elastic effect causes a positive shift in the center frequency. In this study, the resonant frequency of the G-SAW sensor showed a positive shift in the center frequency with an increase in the concentration of acetone gas molecules. Therefore, an elastic effect occurred due to the adsorption of acetone gas molecules onto the surface of the graphene layer.

**Fig. 7:**
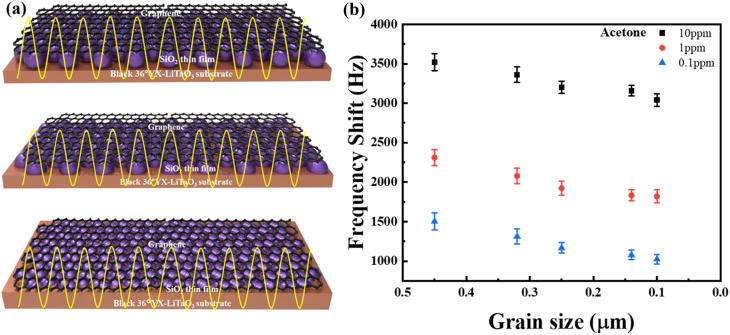
(a) Schematic representation of the G-SAW sensor device with different particle sizes. (b) Effect of sensing response with respect to the grain size of SiO_2_.

Fig. 7 indicates that the gas sensitivity of the G-SAW sensor is highly dependent on the surface roughness of the deposited SiO_2_ thin film. As shown in the schematic image, graphene is suspended between the nanoparticles of the SiO_2_ guiding layer. In the case of suspended graphene, the van der Waals interactions between graphene and the surface atoms of the substrate are removed, leading to enhanced electronic, mechanical, and chemical properties compared to those of non-suspended graphene. In addition, the suspended graphene exhibits fewer wrinkles and crystal defects, thus enhancing the elastic properties and sensitivity of the G-SAW sensor. It has been reported that electron mobility can be increased up to 200 000 cm^2^ V^−1^ s^−1^ and the single molecule can be detected.^47^ When gas molecules are introduced, the graphene membrane undergoes deflection that is approximately two orders of magnitude larger than that of a thin film with lower roughness. The molecules tended to move into a high-propagation area on the graphene surface and lose their kinetic energy. Consequently, molecules can be easily captured by graphene *via* van der Waals (vdW) interactions. In general, gas molecules can easily escape and desorb from the interaction surfaces of graphene. In contrast, when an SAW Love wave is applied, the adsorbed gas molecules can continuously interact with the graphene surface owing to the reduced kinetic energy. The acetone gas molecule showed higher sensitivity than the other gas molecules because of its molecular size and higher strain on the surface of the graphene. It is also evident that a decrease in the roughness of the film leads to a low gas response, as depicted in Fig. 7(b). The charge distribution of acetone molecules can be distorted from their normal shape by propagation on the graphene surface. In addition, the G-SAW sensor showed higher sensitivity towards acetone gas molecules with higher dipole moments (2.88 D), as shown in Table 3. Acetone gas molecules are preferentially adsorbed on CVD monolayer graphene, with O atoms above the hollow site of the C ring. Acetone adsorbed on graphene with a small binding energy enhances the mobility of graphene at room temperature. This resulted in stronger van der Waals interactions with the graphene surface, leading to increased hardness and compressive stress. Furthermore, the high dipole moment of acetone induces stronger electrostatic interactions with graphene, leading to deformation and surface hardness. These results indicate that the elastic effect plays a major role in the high sensitivity toward acetone gas molecules.

**Table 3:** Basic data table of gas molecules^48^

Analytes	Molecular size (Å)	Molecular weight (g mol^−1^)	Dipole moment (D)
Acetone (C_3_H_6_O)	4.6	58.8	2.88
Ethanol (C_2_H_6_O)	4.3	46.07	1.6
Ammonia (NH_3_)	2.3	17.03	1.48

## 5 Conclusion

In this study, a Love-wave-based G-SAW gas sensor was fabricated, and its gas-sensing response towards ammonia and acetone was studied at room temperature. The SiO_2_ guidance plays a major role in noise reduction and insertion loss. The G-SAW showed remarkable sensitivity toward acetone gas molecules at low concentrations (800 ppt) at room temperature. The acetone gas-sensing mechanism of the G-SAW sensor was based on the graphene partially suspended between the nanoparticles and the dipole interactions between the graphene and gas molecules. The G-SAW sensor exhibited a rapid positive shift in the center frequency, which indicated a change in the Young's modulus when the gas molecules were adsorbed onto the graphene surface. Resistance measurements confirmed the elastic effect of the G-SAW gas sensor.

## Conflicts of interest

The authors declare that they have no known competing financial interests or personal relationships that could have appeared to influence the work reported in this study.

## Supplementary Material

NA-005-D3NA00914A-s001
