# Supplementary material for: Real-time detection of acetone gas molecules at ppt levels in an air atmosphere using a partially suspended graphene surface acoustic wave skin gas sensor
Source: Nanoscale Adv. 2023 Nov 17;5(24):6999–7008. doi: 10.1039/d3na00914a (PMC10696977; doi:10.1039/d3na00914a)

Electronic Supplementary Material (ESI) for Nanoscale Advances.
This journal is © The Royal Society of Chemistry 2023

Supplementary file

Real-Time Detection of Acetone Gas Molecules at ppt Levels in Air Atmosphere Using a Partially Suspended Graphene Surface Acoustic Wave Skin Gas Sensor

Haolong Zhou [a], Sankar Ganesh Ramaraj [a, b *], Kaijie Ma [b], Md Shamim Sarker [a], Zhiqiang Liao [a], Tang Siyi [a], Hiroyasu Yamahara [a *], Hitoshi Tabata [a, b *]

[a] Department of Electrical Engineering and Information Systems, Graduate School of Engineering, The University of Tokyo, 7-3-1 Hongo, Bunkyo-ku, Tokyo 113-8656, Japan

[b] Department of Bioengineering, Graduate School of Engineering, The University of Tokyo, 7-3-1 Hongo.

Corresponding author email: Hitoshi Tabata: tabata@g.ecc.u-tokyo.ac.jp; Hiroyasu Yamahara: yamahara@bioxide.t.u-tokyo.ac.jp; Sankar Ganesh Ramaraj: ramaraj@g.ecc.u-tokyo.ac.jp

Figure S1 (a) Surface roughness of different thickness of SiO2 film, (b) Grain size of SiO_2 (3 μm) film grown at different temperature.

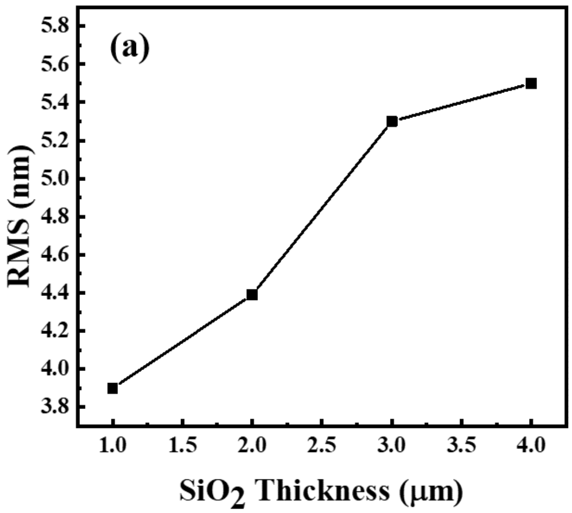

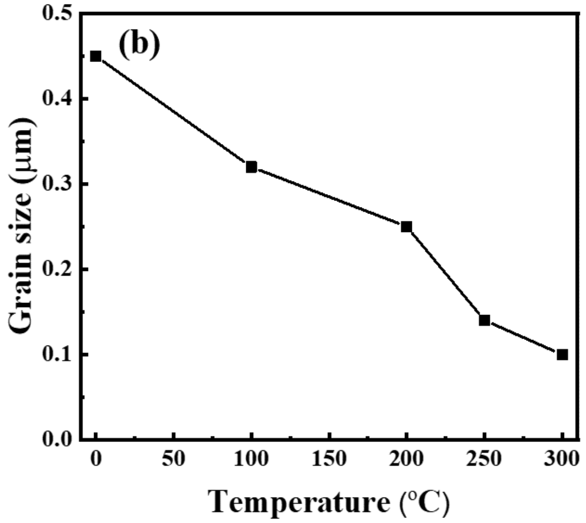

Figure S2 AFM image of 3 μm SiO_2 film deposited at different temperatures, (a).100 °C, (b). 200 °C, (c). 250 °C, (d). 300 °C respectively.

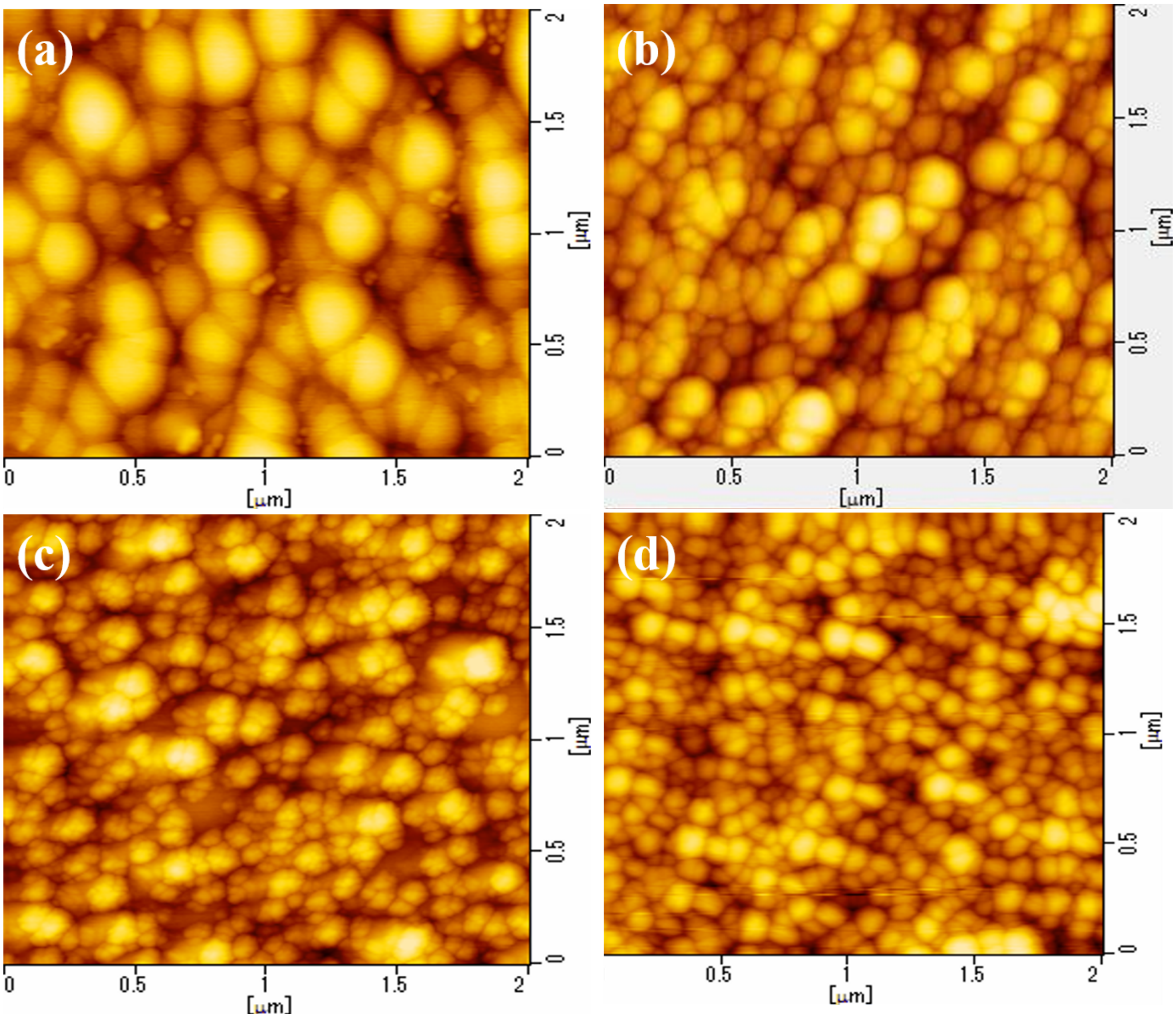

Figure S3 Surface acoustic wave spectra of 1, 2, 3 & 4 μm deposited SiO_2 thin film.

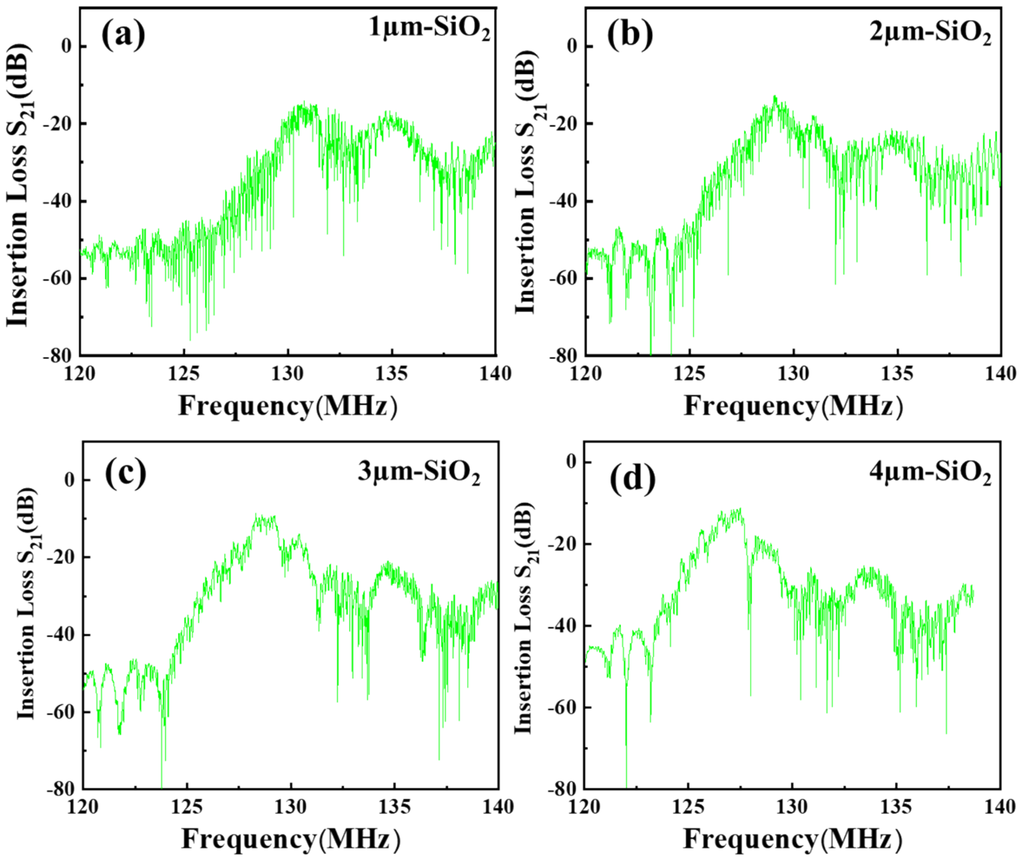

Figure S4 Dynamic frequency response curves of the G-SAW sensor towards (a). acetone, (b). Ammonia, & (c). Ethanol and gas molecules, respectively.

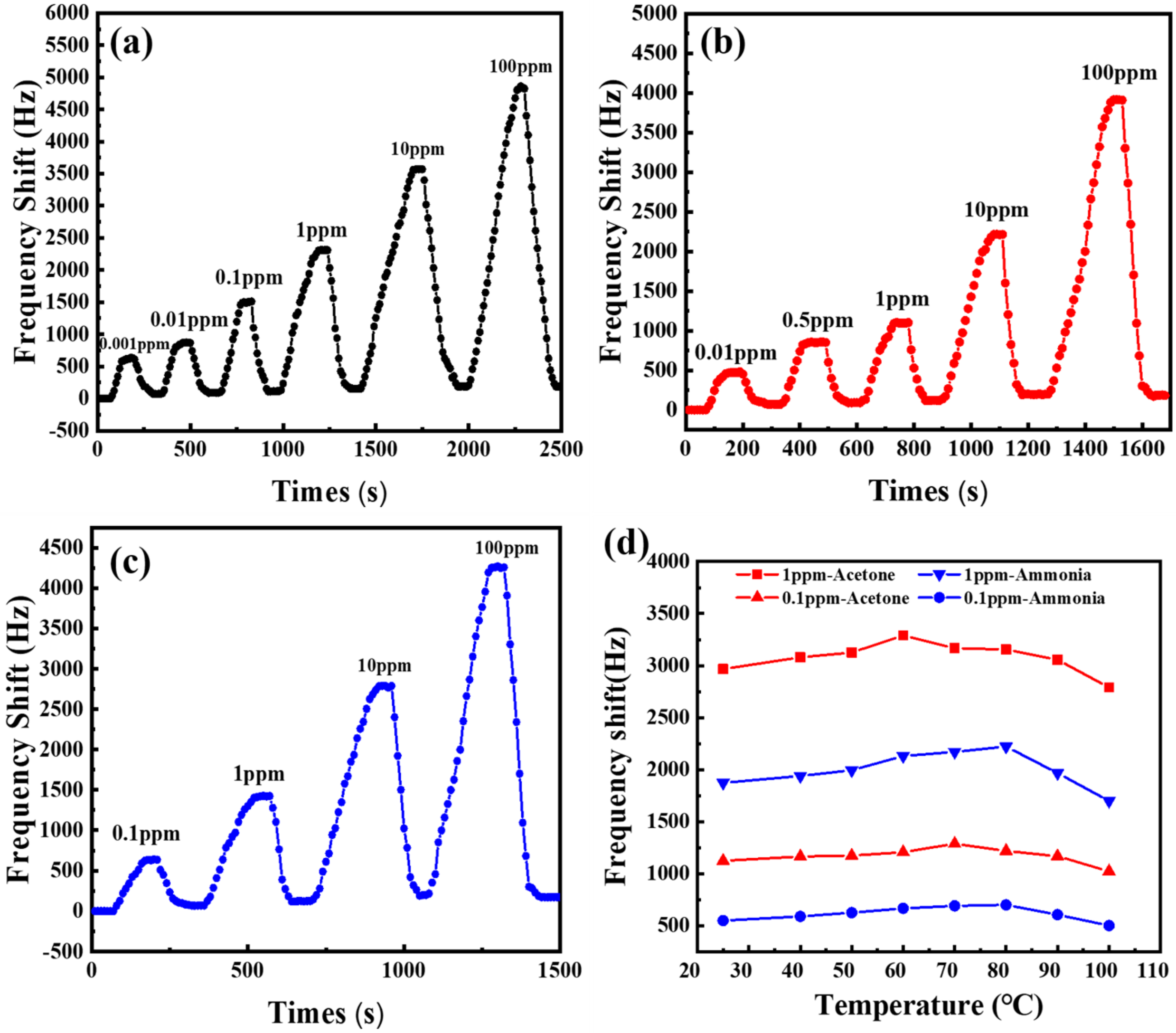

Figure S5. Gas Response of graphene SAW sensor towards acetone under different humidity and temperature.

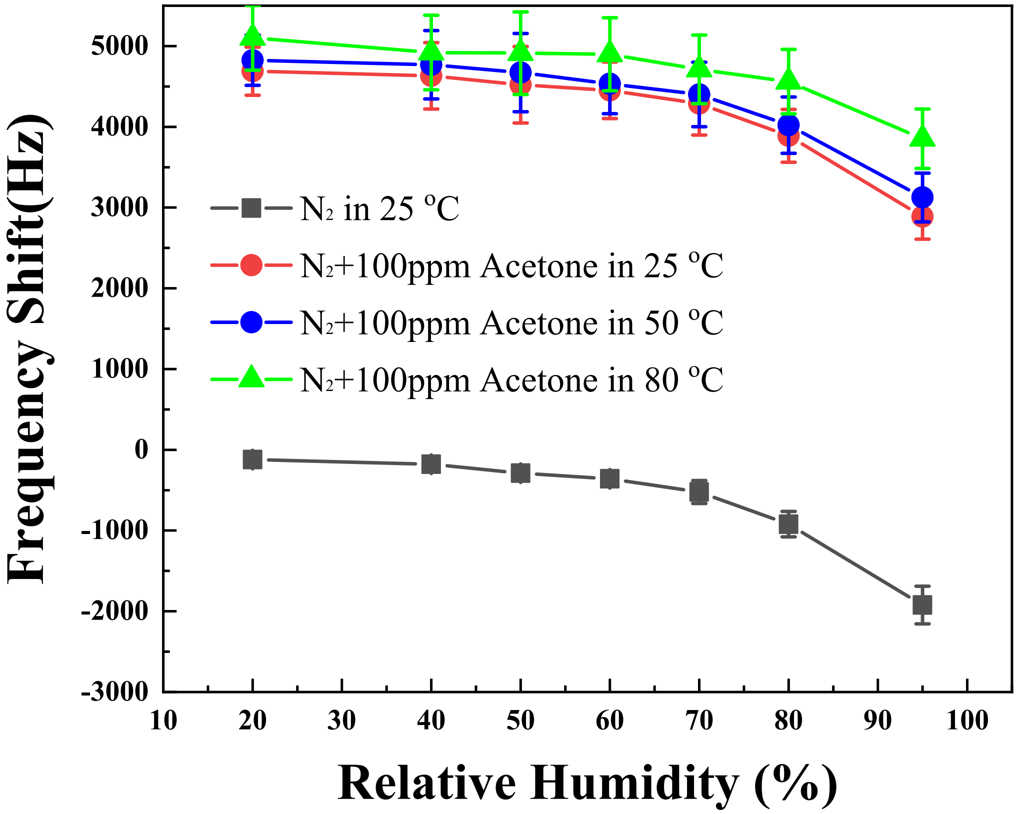

Supplement: NA-005-D3NA00914A-s001 [file NA-005-D3NA00914A-s001.pdf]
